# Autophagy Is Independent of the Chondroprotection Induced by Platelet-Rich Plasma Releasate

**DOI:** 10.1155/2018/9726703

**Published:** 2018-07-24

**Authors:** Fan Yang, Haoran Hu, Wenjing Yin, Guangyi Li, Ting Yuan, Xuetao Xie, Changqing Zhang

**Affiliations:** Department of Orthopedic Surgery, Shanghai Jiao Tong University Affiliated Sixth People's Hospital, 600 Yishan Road, Shanghai 200233, China

## Abstract

**Background:**

Platelet-rich plasma (PRP) has been shown to be a promising therapeutic agent against osteoarthritis (OA), whereas its chondroprotection mechanism is not fully elucidated. Autophagy is considered an important biological process throughout the development of OA. Therefore, the objective of the present study is to investigate the role of autophagy in the chondroprotection and compare the effects of releasate between L-PRP and P-PRP.

**Methods:**

PRP were prepared from rat blood. Rat chondrocytes pretreated in the presence or absence of interleukin-1 beta (IL-1*β*) were incubated with PRP releasate. The expressions of OA-related genes and autophagy-related genes were determined by RT-PCR and western blot, respectively. Autophagic bodies were assessed by transmission electron microscopy and the autophagy flux was monitored under the confocal microscopy. The effect of PRP on autophagy was further investigated in the milieu of autophagy activator, rapamycin, or autophagy inhibition by downregulation of Atg5. The effect of PRP on cartilage repair and autophagy was also evaluated in an OA rat model.

**Results:**

* In vitro*, PRP releasate increased the expression of the anabolic genes, COL2 and Aggrecan, and decreased the expression of the catabolic genes, whereas the expression of autophage markers, Atg5 and Beclin-1, as well as the ratio of LC3 II/LC3 I, was not significantly altered in normal or IL-1*β*-treated chondrocytes. Similar expression pattern was found following the activation (rapamycin) or inhibition (Atg5 silencing) of autophagy.* In vivo*, PRP releasate ameliorated posttraumatic cartilage degeneration while the expression of LC3 was comparable to that in the vehicle treatment group.

**Conclusions:**

PRP releasate promoted the anabolic gene expression, relieved inflammatory stress in chondrocytes, and ameliorated cartilage degeneration, but autophagy was independent of these processes.

## 1. Background

Osteoarthritis (OA) is a multifactorial disease which is characterized by degeneration of the joints [1]. Destruction of articular cartilage is a critical event for OA initiation and progression [2], and it was mainly attributed to impaired chondrocyte function and the imbalanced homeostasis in joints [3]. A number of signals and molecules have been investigated to develop anti-OA therapies; however, current clinical trials failed to provide conclusive evidence of their efficacy [4, 5]. A possible explanation is that these therapeutic approaches are highly specific and thus fail to constitute sufficient impact on the overall disease or preclude cartilage degeneration.

Platelet-rich plasma (PRP), isolated from autologous blood, is a plasma preparation containing a higher concentration of platelets. The platelets secret multiple kinds of growth factors, such as transforming growth factor *β* (TGF-*β*), platelet-derived growth factor (PDGF), insulin-like growth factor, basic fibroblast growth factor, and vascular endothelial growth factor [6, 7]. PRP has been proved to be able to promote chondrocyte proliferation and secretion of extracellular matrix. Besides the anabolic effect, PRP has inhibitory impact on the inflammatory process in osteoarthritic chondrocytes [8, 9]. Due to the repertoire of cytokines, one would infer that PRP might target a number of key signaling pathways involved in the OA pathogenesis and improve the imbalanced homeostasis of the degenerated joints. PRP has been increasingly used in the treatment of OA in the past decade [10]. A meta-analysis of randomized controlled trials revealed the efficacy of PRP containing concentrated leucocytes (Leucocyte-rich PRP, L-PRP) or not (Pure PRP, P-PRP) in the improvement of knee pain and function in OA patients [11], while the underlying mechanism of PRP has yet to be elucidated.

Autophagy is an essential cellular homeostasis mechanism. Its modulation is integrated with multiple signal transduction pathways that respond to inflammatory cytokines, growth factors, nutrient supply, and energy balance [12, 13]. Recently, studies revealed that autophagy plays a critical role in the pathogenesis of OA [13–15]. In particular, it has a protective role in human chondrocytes and prevents chondrocytes from undergoing OA changes [16]. The activation of autophagy is perceived as an adaptive response to protect chondrocytes from various stresses, including excessive inflammation and nutrient deprivation [13]. The failure of this response may lead to cartilage degeneration [15].

Therefore, the present study aimed to determine whether the releasate of PRP exhibited chondroprotective effect via the modulation of autophagy in chondrocytes.

## 2. Methods

### 2.1. PRP Preparation

Eight-week-old male SD rats (200 ± 20 g; Silaike, Shanghai, China) were used to harvest blood samples and prepare PRPs. The rats were kept in SPF conditions according to the guidelines of Animal Care of Shanghai Jiaotong University School of Medicine and acclimatized for one week before use.

After anesthesia, approximately 10 mL of whole blood (WB) was collected from the central auricular artery of each rat. Blood coagulation was inhibited by anticoagulant Citrate Dextrose Solution A (ACD-A; Turner, USA). The anticoagulated WB was used to prepare PRP.

#### 2.1.1. L-PRP Preparation

In brief, the anticoagulated WB was centrifuged at a speed of 1800 rpm for 10 min in order to separate the blood into three layers: a bottom layer of red blood cells (RBCs), a middle buffy coat layer containing leukocytes, and a top plasma layer containing platelets. The top and middle layers were transferred to a new tube and centrifuged at a speed of 3000 rpm for 10 min. Then supernatant was discarded; the pellet containing concentrated platelets and leukocytes was resuspended to obtain 1 ml of L-PRP.

#### 2.1.2. P-PRP Preparation

The anticoagulated WB was centrifuged at a speed of 1000 rpm for 10 min. The top plasma layer was carefully collected and then centrifuged at a speed of 3000 rpm for 10 min. The supernatant was discarded and precipitated platelets were resuspended to obtain 1 ml of P-PRP.

Thrombin (1000 U in 10% CaCl_2_; 9:1) was added to L-PRP or P-PRP and then incubated at 37°C in a humidified atmosphere containing 5% CO_2_ for 30 min. After incubation, the sample was centrifuged at 4000 rpm for 15 min. The supernatant was designated as L-PRP or P-PRP releasate, which were used for the subsequent experiments.

### 2.2. Component and Cytokine Analysis of PRP

The concentrations of platelets and leukocytes in the WB, unactivated L-PRP and P-PRP, were measured using an automated hematology analyzer (XS-800i™; Sysmex, Kobe, Japan). The levels of interleukin 1-*β* (IL-1*β*, anti- rat), tumor necrosis factor-*α* (TNF-*α*, anti- rat), PDGF-AB (anti- rat), and TGF-*β*1 (anti- rat) in the L-PRP or P-PRP releasate were detected by enzyme linked immunosorbent assay (ELISA) assay according to the manufacturer's instructions (Xitang Biological Technology Ltd., Shanghai, China).

### 2.3. Chondrocyte Isolation and Culture

After rats were sacrificed, articular cartilages of the femoral heads were peeled, cut into small pieces, and digested with 0.3% collagenase for 12 h at 37°C. Then, the suspension containing the chondrocytes was centrifuged at a speed of 1500×g for 10 min, and the chondrocyte pellet was washed with PBS, resuspended in Dulbecco's Modified Eagle's Medium/F-12 (DMEM/F-12) medium (Gibco BRL, Grand Island, NY, USA), which contains 10% (v/v) of fetal calf serum (FCS, Gibco, USA) and 1/100 streptomycin-penicillin (Gibco) and maintained in humidified atmosphere containing 5% CO_2_ at 37°C. Three to six passages of chondrocytes were used for further experiments.

#### 2.3.1. PRP Releasate Treatment

Releasate of L-PRP or P-PRP at the concentration of 2% or 10% were added to the chondrocyte culture medium. After incubation for 24 h or 96 h, chondrocytes were collected for analysis.

#### 2.3.2. IL-1*β* Treatment

In order to mimic the inflammatory stimuli in OA, chondrocytes were incubated with 10 ng/ml IL-1*β* 2 h prior to PRP treatment [17]. Chondrocytes in the control group were treated with vehicle solution (0.1 M PBS).

#### 2.3.3. Autophagy Activation

Autophagy was activated by rapamycin treatment. In brief, a number of 5 × 10^5^ chondrocytes were plated in 6-well plates and incubated with 10 *μ*M rapamycin for 24 or 96 hours.

#### 2.3.4. Autophagy Inhibition

Autophagy was inhibited via silencing the expression of Atg5. In brief, complementary oligonucleotides sequences (5′-CCTGTTTACAGTCAGTCTATT-3′) against Atg5 or scramble sequences were synthesized and cloned into pLLU2G lentiviral vectors to construct pLLU2G-CyPA small hairpin RNA (shRNA) plasmids. Lentivirus was produced by the transient transfection of shRNA plasmids in HEK-293T cells. The stable transfection procedure was the same as mRFP-GFP-LC3 transfection. Lentivirus transfected cells were then selected in the presence of 1.5 *μ*g/ml puromycin for three days to obtain stably transfected chondrocytes, then cells were maintained in 0.5 *μ*g/ml puromycin. The efficiency of the Atg5 silencing was determined by western blot.

### 2.4. Real-Time PCR (RT-PCR)

After due treatment, total RNA was extracted from chondrocytes using TRIzol® reagent (Invitrogen, Carlsbad, CA, USA) according to the manufacturer's instructions. Then RNA was reverse transcribed to cDNA according to the manufacturer's protocols (Takara, Kyoto, Japan). RT-PCR was subsequently performed using SYBR® Premix Taq (Takara) and a Thermal Cycler Dice real-time TP800 system (Takara). The primers of Collagen II (COL2), Aggrecan, matrix metalloproteinase (MMP) 13, and a disintegrin and metalloproteinase with thrombospondin motifs (ADAMTS) 5 were listed in Table 1. The targeted genes were analyzed by the 2^-ΔΔCt^ method and GAPDH was used as a reference gene.

### 2.5. Western Blot Analysis

After treatment, protein was extracted using RIPA lysis buffer (Beyotime, China). Protein concentration was measured by Bradford assay, then equal amounts of protein (15 *μ*g/lane) were separated by SD-polyacrylamide gels (SDS-PAGE, 7-15%) and transferred onto PVDF membranes (Millipore, USA). Nonspecific signals were blocked by 5% (w/v) skim milk, then membranes were incubated with primary antibodies against LC3A/B (Cell Signaling Technology, 1:1000), Atg5 (Cell Signaling Technology, 1:1000), Beclin-1 (Cell Signaling Technology, 1:1000), and GAPDH (Cell Signaling Technology, 1:1000) overnight at 4°C. Afterwards, corresponding secondary antibodies were incubated for 2 h at room temperature in dark. Protein bands were measured by electrochemiluminescence (Bio-Rad, USA).

### 2.6. Transmission Electron Microscopy (TEM)

A number of 1 × 10^5^ cells were seeded on coverslips, after 24 treatment with PRP releasate, the cells were washed in phosphate-buffered saline (PBS, pH 7.4). Afterwards, cells were fixed in 0.2% glutaraldehyde/PBS for 2 h at room temperature, postfixed in 1% osmium tetroxide in distilled water for 1 h, and then stained with 2% uranyl acetate in water for 1 h in the dark. After dehydration in a gradient series of ethanol, the cells were embedded in Epon-Araldite resin. Embedded blocks were cut into 80-nm thick ultrathin sections. Sections were then mounted onto Formvar®-coated copper mesh grids, counterstained with uranyl acetate and lead citrate, and finally observed under a TEM (Philips; Eindhoven, the Netherlands).

### 2.7. mRFP-GFP-LC3 Transfection

About 1.5 × 10^5^ chondrocytes were seeded in 6-well plates in antibiotics deprivation medium overnight. When the confluence reached approximately 70%, chondrocytes were transfected with mRFP-GFP-LC3 (HanBio Technology Co. Ltd.; Shanghai, China) or the vehicle according to manufacturer's protocols. Then cells were cultured for 2 h and then 2% fetal bovine serum was added. The transfection efficacy was measured by fluorescence microscope (Leica TCS SP8; Leica Microsystems, Wetzlar, Germany). After mRFP-GFP-LC3 stable transfected chondrocytes were constructed, autophagosomes are visualized as yellow dots, and autolysosomes are red dots under a confocal microscope (Leica TCS SP8 STED 3X;). At 24 h following PRP releasate treatment, the number of autophagosomes and autolysosomes was calculated.

### 2.8. Experimental OA in Rats

Thirty 12-week-old SD rats (250 ± 15 g) were adopted for the establishment of an experimental OA model [18]. On the first day of the study, rats were anesthetized, and the medial meniscus of the right hind limb was cut to simulate a complete tear. Three weeks later, the rats' right hind limb were intra-articularly injected a volume of 50 *μ*L releasate of L-PRP, P-PRP, or PBS once a week for 3 weeks. Sham-operated control rats were intra-articularly injected with PBS once a week for 3 weeks. The cartilage degeneration score was assessed in each representative section using as previously described [19]. It is based on cartilage matrix loss, chondrocyte loss/death, and proteoglycan loss and ranges from 0 (no cartilage degeneration) to 5 (severe degeneration, with greater than 75% cartilage loss).

### 2.9. Histological Analysis

After rats were sacrificed, the tibiofemoral joint was separated from the hind limb. The joints were then fixed in 4% formaldehyde for at 4°C for 72 h, decalcified in 10% formic acid for 10 days, and embedded in paraffin. The joint was sectioned at 5-*μ*m thick, then mounted onto glass slides, deparaffinized, and stained with Safranin O. The remaining sections were dried overnight and used for immunohistochemical analyses.

### 2.10. Statistical Analysis

Data are presented as means ± standard deviation. Differences between groups were assessed by one-way analysis of variance (ANOVA) followed by a post hoc Tukey's test. Comparisons between two groups were assessed by unpaired, two-tailed t-tests after evaluating the data distribution for equal variance using an F-test. All statistical analyses were performed using SPSS 22.0 (SPSS, IBM, USA). A two-tailed p-values less than 0.05 were considered statistically significant.

## 3. Results

### 3.1. Components and Cytokines in PRP

**Figure 1:**
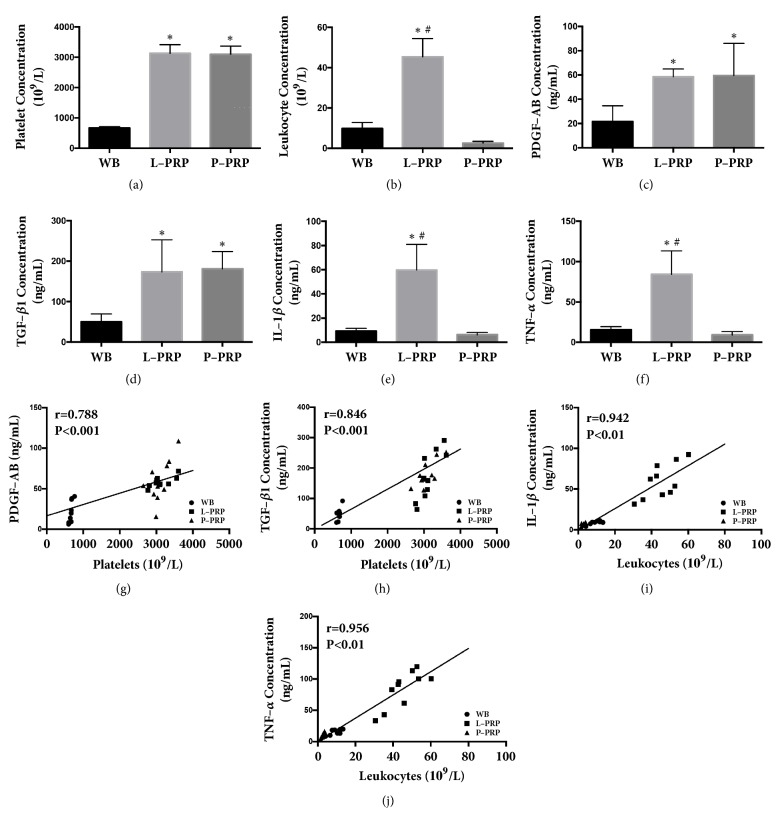
**Analysis of components and cytokines in the whole blood, P-PRP, and L-PRP**. Platelet and leukocyte concentrations were measured using an automated hematology analyzer; growth factors were measured by ELISA. Platelet (a), PDGF-AB (c), and TGF-*β*1 (D) concentrations in L-PRP and P-PRP were comparable, but significantly higher than those in WB; leukocyte (b), IL-1*β* (e), and TNF-*α* (f) concentrations in P-PRP were significantly lower than those in WB and L-PRP. The platelet count was positively correlated with the PDGF-AB (g) and TGF-*β*1 (h) levels in P-PRP; the leukocyte concentration was positively correlated with the IL-1*β* (i) and TNF-*α* (j) concentrations. ELISA, enzyme linked immunosorbent assay. *∗* represents significant difference versus WB; # represents significant difference versus P-PRP.

Compared with WB, the count of platelets was significantly higher in L-PRP and P-PRP (Figure 1(a)), whereas no significant difference was observed between L-PRP and P-PRP. The count of leukocytes was significantly higher in L-PRP, compared with that in WB and P-PRP, whereas there is no significant difference between WB and P-PRP. Moreover, the releasate of L-PRP contained higher level of IL-1*β* and TNF-*α* than that in WB and the releasate of P-PRP contained higher level of PDGF-AB and TNF-*α* (all p < 0.05; Figures 1(c)-1(f)).

Furthermore, correlation analysis demonstrated that the platelet count was positively correlated with the PDGF-AB and TGF-*β*1 concentration (PDGF-AB: r = 0.788, p < 0.001; TGF-*β*1: r = 0.846, p < 0.001) (Figures 1(g) and 1(h)). In addition, the leukocyte concentration was positively correlated with the level of IL-1*β* and TNF-*α* (IL-1 *β*: r = 0.942, p < 0.01; TNF-*α*: r = 0.956, p < 0.01) (Figures 1(i) and 1(j)). These results indicate that the components and cytokines in L-PRP and P-PRP were differed.

### 3.2. PRP Releasate Modulated OA-Related Gene Expression but Did Not Alter the Autophagy-Related Gene Expression

**Figure 2:**
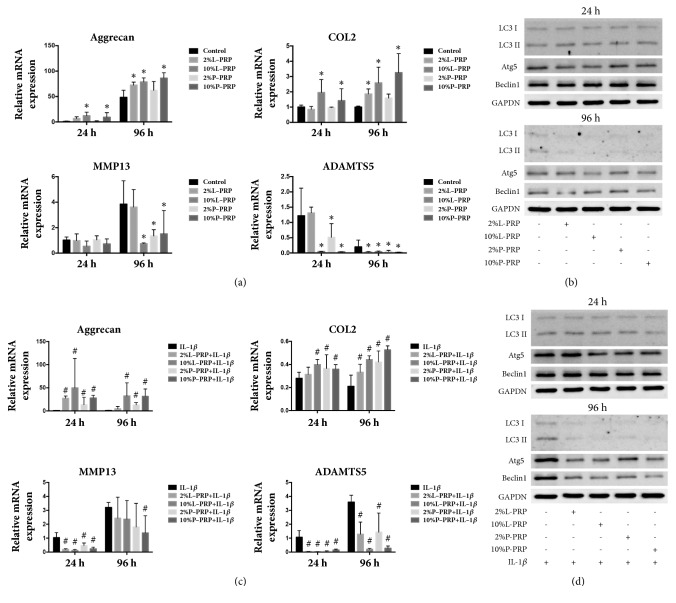
**Effect of PRP on normal chondrocytes or IL-1**β**-treated chondrocytes**. The chondrocytes were cocultured with either 2% or 10% L-PRP or P-PRP for 24 or 96 h. (a, c) The expression of cartilage-related genes (Aggrecan, COL2, ADAMTS5, and MMP13) at the mRNA level in chondrocytes treated without (a) or with IL-1*β* (c). (b, d) Western blot analysis for autophagy-related proteins: LC3 II/LC3 I, Atg5, and Beclin 1. *∗* represents significant difference versus control group; # represents significant difference versus IL-1*β* group.

In chondrocytes without the presence of IL-1*β*, as shown in Figure 2(a), releasate of both L-PRP and P-PRP (both 2% and 10% concentration) significantly upregulated the expression of Aggrecan and COL2 at 24 and 96 h, in comparison with the control group without PRP releasate treatment (all p < 0.05). The expression of the ADAMTS5 was lowered at both time points and MMP13 was decreased at the 96 h following PRP releasate (both 2% and 10% dilution) treatment (Figure 2(a)). Whereas the expression of autophagy-related genes such as Atg5 and Beclin1, as well as the ratio of LC3 II/LC3 I at the protein level, was not significantly affected after both types of PRP releasate treatment (both 2% and 10% concentration) at both time points (Figure 2(b)).

When chondrocytes were treated with IL-1*β*, the releasate of L-PRP and P-PRP (both 2% and 10% concentration) increased the mRNA expression of Aggrecan and COL2, whereas the mRNA expression of MMP13 and ADAMTS5 was decreased at both time points in comparison with the control group (all p < 0.05, Figure 2(c)). After treatment of L-PRP and P-PRP releasate, the expression of Beclin1, Atg5, and the ratio of LC3 II/LC3 I at the protein level were unaffected at the 24 h and 96 h time points (Figure 2(d)). These results suggest that autophagy may not be affected by the releasate of L-PRP or P-PRP treatment in their role of chondroprotection.

### 3.3. The Autophagic Flux Was Not Affected by PRP Releasate

**Figure 3:**
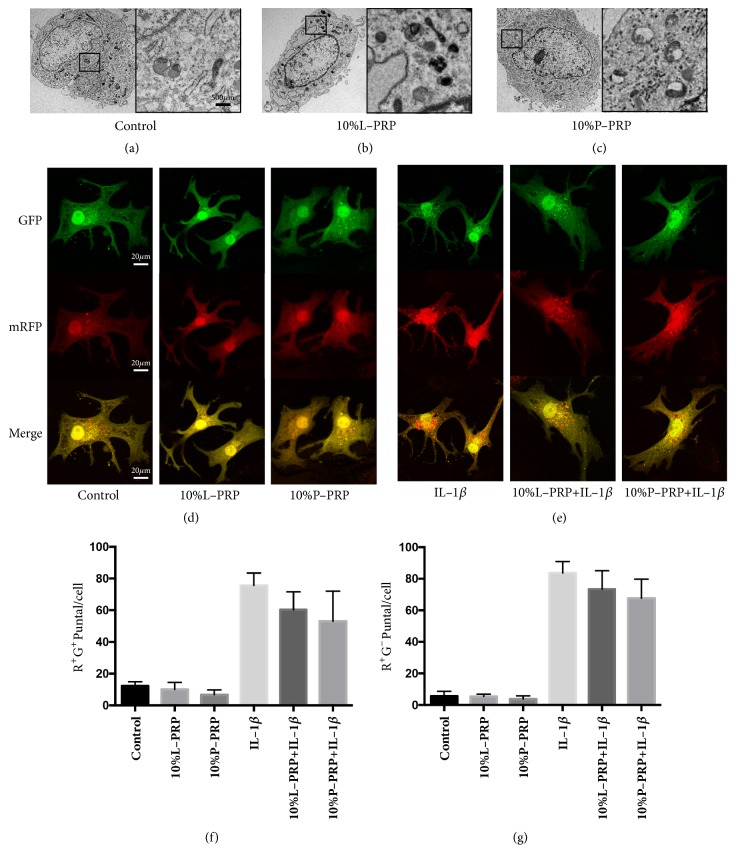
**Effect of PRP on the autophagy flux of normal chondrocytes or IL-1**β**-treated chondrocytes.** (a, b, and c) Transmission electron microscopy (TEM) analysis the ultrastructure of control (a), releasate of 10% L-PRP-treated (b) and 10% P-PRP-treated (c) chondrocytes. Chondrocytes were transfected with mRFP-GFP-LC3 to follow autophagic flux, and then they were treated with or without IL-1*β* and exposed to releasate of 10% L-PRP or 10% P-PRP. (d, e) Confocal images of labeled autophagosomes (yellow) and autolysosomes (red). The number of autophagosomes was quantified (f), and the number of autolysosomes was quantified (g). Data are represented by means ± standard deviation.

In chondrocytes without the presence of IL-1*β*, the autophagosomes were observed in the control treatment and the groups treated by L-PRP or P-PRP releasate at 24 h (Figures 3(a)-3(c)). With the transfection of mRFP-GFP-LC3, the number of autophagosomes (yellow dots) and autolysosomes (red dots) was counted, respectively. After exposure to L-PRP or P-PRP releasate for 24 h, there was no significant change in the number of both autophagosomes and autolysosomes in comparison with the control group (Figures 3(d)-3(g)). Under the circumstance of IL-1*β* addition, while L-PRP and P-PRP releasate reduced the number of autophagosomes and autolysosomes, the difference was not statistically significant (Figure 3(g)), indicating that PRP releasate did not change the autophagic flux.

### 3.4. Effect of PRP on Normal Chondrocytes or IL-1*β*-Treated Chondrocytes with Autophagy Activation

**Figure 4:**
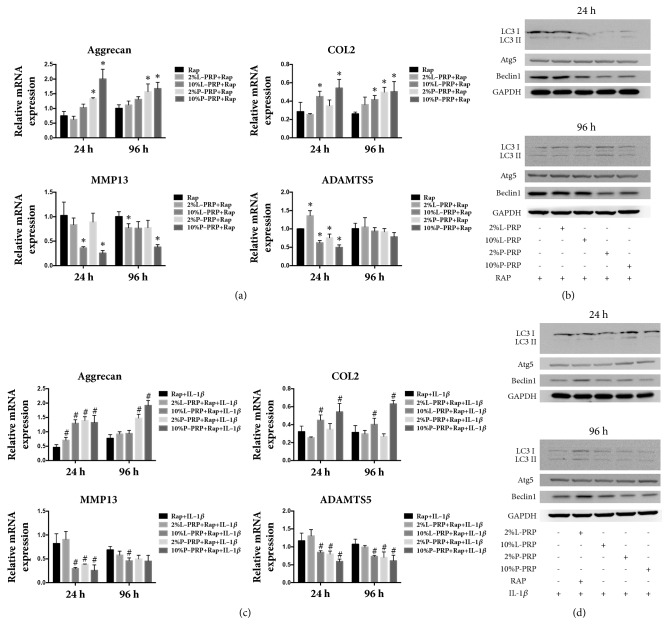
**Effect of PRP on normal chondrocytes or IL-1**β**-treated chondrocytes with autophagy activation**. (a, c) The expression of Aggrecan, COL2, ADAMTS5, and MMP13 in chondrocytes was measured by RT-PCR. (b, d) The expression of LC3 II/LC3 I, Atg5, and Beclin 1 was analyzed by western blot. *∗* represents significant difference versus control group; # represents significant difference versus IL-1*β* group.

Upon autophagy activation by rapamycin, the mRNA expression of Aggrecan and COL2 was increased at 24 h and 96 h after L-PRP or P-PRP releasate treatment; the expression of ADAMTS5 and MMP13 was significantly decreased at 24 h (Figure 4(a)). Although the protein expression of Beclin-1 after P-PRP releasate treatment was decreased at both time points, no significant change was observed in terms of the expression of Atg5 and the LC3 II/LC3 I ratio after either L-PRP or P-PRP releasate treatment (Figure 4(b)). Meanwhile, similar expression patterns were observed in IL-1*β*-treated chondrocytes after rapamycin treatment with regard to cartilage-promoting genes, in which the expression of COL2 and Aggrecan were upregulated (Figure 4(c)). The expression of autophagy-related genes at the protein level were comparable before and after releasate of L-PRP- or P-PRP treatment (Figure 4(d)).

### 3.5. Effect of PRP on Normal Chondrocytes or IL-1*β*-Treated Chondrocytes with Autophagy Inhibition

**Figure 5:**
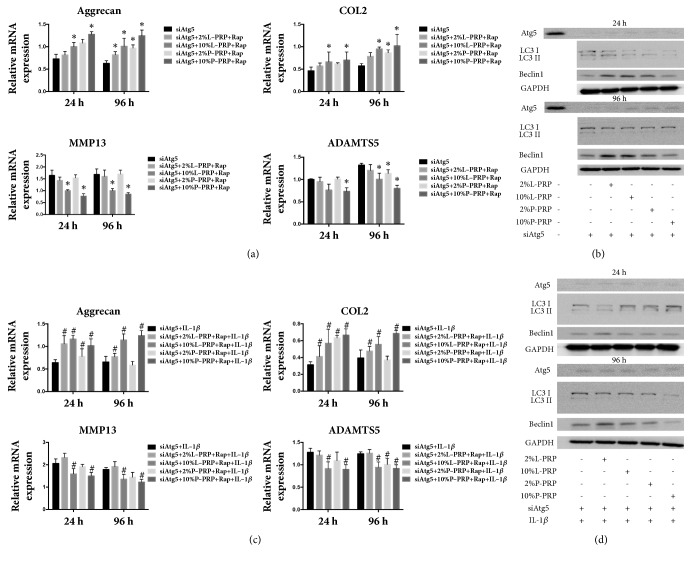
**Effect of PRP on normal chondrocytes or IL-1**β**-treated chondrocytes with autophagy inhibition**. (a, c) The expression of Aggrecan, COL2, ADAMTS5, and MMP13 was measured by RT-PCR. (b, d) The expression of LC3II/LC3I, Atg5, and Beclin 1 was measured by western blot. *∗* represents significant difference versus control group; # represents significant difference versus IL-1*β* group.

After shRNA transfection of Atg5, the expression of Atg5 was significantly decreased at the protein level (Figure 5(b)). The expression of Aggrecan and COL2 was significantly upregulated after L-PRP or P-PRP releasate treatment of chondrocytes, both in the absence and in presence of IL-1*β* at the two time points (Figures 5(a) and 5(c)). Consistent with observations in rapamycin treatment, the expression of ADAMTS5 and MMP13 was decreased at both time points after L-PRP or P-PRP releasate treatment (Figures 5(a) and 5(c)). Nevertheless, the expression of Atg5, LC3 II/LC3 I, and Beclin1 was not significantly impacted both in the presence and in absence of IL-1*β* (Figures 5(b) and 5(d)).

### 3.6. Effect of PRP on Traumatic Injury of Articular Cartilage In Vivo

**Figure 6:**
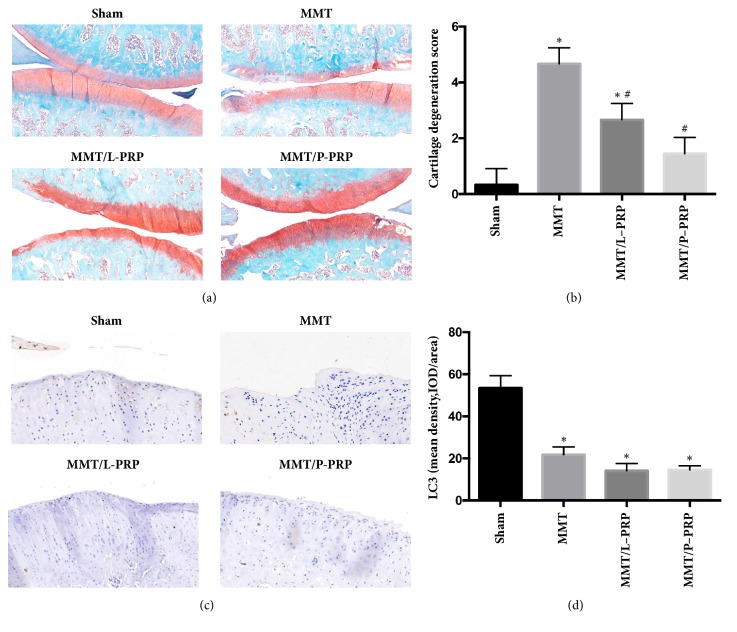
**Effect of PRP on traumatic injury of articular cartilage* in vivo***. Three weeks after the cut of medial meniscus, rats (8 in every group) were received intra-articular injection of L-PRP or P-PRP releasate once a week for 3 weeks. (a) Representative images of tissue sections through the tibiofemoral joint stained with the Safranin O. Images were acquired at a magnification of 100x. Scale bars: 500 *μ*m. (b) Histograms showing the mean cartilage degeneration scores of the sham, MMT, and PRP-treated joints. (c, d) The expression of LC3 was assessed by immunochemical staining. *∗* represents significant difference versus the sham group; # represents significant difference versus the MMT group. MMT: medial meniscus tear.

Medial meniscus tear induced severe cartilage degeneration on the medial tibia plateau compared with rats in the sham group as indicated by Safranin O staining. However, cartilage degeneration was alleviated after L-PRP or P-PRP releasate treatment as indicated by Safranin O staining (Figures 6(a) and 6(b)). Moreover, the expression of LC3 was significantly decreased after medial meniscus tear, whereas its expression was comparable before and after L-PRP or P-PRP releasate treatment (Figures 6(c) and 6(d)). These results indicate that autophagy may be unassociated with the chondroprotection induced by the releasate of L-PRP and P-PRP in OA.

## 4. Discussion

In this study, we evaluated the role of autophagy during the PRP releasate treatment in the normal chondrocytes, IL-1*β*-treated chondroctyes, and experimental OA knee joints. Results showed that PRP promoted anabolic gene expression, inhibited inflammatory stress, and attenuated posttraumatic cartilage degeneration, while the autophagy level did not differ significantly between before and after PRP releasate treatment* in vitro* or* in vivo*, even under the circumstances of increased autophagy by rapamycin or inhibited autophagy by shRNA transfection.

In the past decade, PRP has been widely used in sports injury therapy due to its beneficial effects in tissue repair [20, 21], whereas the preparation methods may result in varied concentration of platelets and leukocytes which, in turn, affect circulating cytokine levels. It was suggested that TNF-*α* and IL-1*β*, which are mainly derived from leukocytes, and circulating GFs and cytokines are associated with the catabolic state in OA [22]. In the present study, L-PRP had a higher concentration of leukocytes, IL-1*β*, and TNF-*α* compared to P-PRP. Compared with WB, the concentration of platelet, PDGF and TGF-*β*1 were significantly higher in the releasate of L-PRP and P-PRP. Moreover, compared with P-PRP, the content of IL-1*β* and TNF-*α* was higher in L-PRP, which may at least in part attribute to that releasate of P-PRP performed better than that of releasate of L-PRP in cartilage degeneration score improvement, as IL-1*β* is well-known cytokine to induce OA in vitro [17].

Autophagy is a well-conserved cell survival mechanism and has been suggested to play important roles in the pathogenesis of OA [13–16]. In the present study, we first examined the effect of 2 distinctive types of PRP releasate at 2 different concentrations on autophagy in normal chondrocytes. No significant changes in terms of the autophagy markers, Atg 5 and Beclin 1, and the ratio of LC3 II/LC3 I were noted at 24 h or 96 h after treatment. Moreover, in order to mimic an osteoarthritic milieu, the effect of PRP on the autophagy activity in IL-1*β*-treated chondrocytes was also evaluated. Sasaki et al. reported that autophagy was increased in chondrocytes under the catabolic stress and rapamycin further increase the level of autophagy [13]. Though PRP is similar to rapamycin in the amelioration of IL-1*β*-induced inflammatory gene expression in vitro and posttraumatic cartilage degeneration in vivo, PRP did not alter the autophagy level in IL-1*β*-treated chondrocytes, indicating that autophagy may not be involved in the chondroprotection induced by PRP releasate.

Furthermore, we examined the autophagy activity during the PRP treatment of chondrocytes on the basis of increased autophagy induced by rapamycin or inhibited autophagy by shRNA transfection. Both the Atg5 expression and the ratio of LC3 II/LC3 I were not significantly affected by the PRP releasate, which further confirmed that PRP treatment is independent of autophagy. A potential reason may be associated with the rich growth factors in L-PRP or P-PRP releasate. Autophagy is perceived as an adaptive response to inflammatory stress or starvation [13]. PRP releasate, on the one hand, provided rich nutrition for chondrocytes to avoid the autophagy increase, but, on the other, PRP did not affect the autophagic response of chondrocytes to the inflammatory stress, which is common in the milieu of knee OA.

These results are interesting, as a number of pharmaceuticals exhibiting chondroprotection effect, such as rapamycin, Torin 1, and glucosamine, could increase the autophagy activity [23–25]. However, PRP may be an exception. It could stimulate the anabolic metabolism and inhibit the inflammatory stress but did not overall affect the autophagy state. Therefore, it may be used under the circumstances that the change of autophagy activity may not be allowed.

To our knowledge, this study is the second study investigating the effect of PRP on the autophagy activity of articular chondrocytes. Conflicting observations with our study, Moussa et al. reported that PRP, probably L-PRP according to their description of PRP preparation method, upregulated the expression levels of Beclin 1 and LC3 II mRNA and increased the number of autophagosomes detected by flow cytometry in human osteoarthritic chondrocytes [26]. The inconsistency with our findings may be attributed to the difference in the tools of autophagy measurement. Our study evaluated the expression of a series of autophagy makers, including Beclin 1 and LC3 II, at the protein level. The turnover of LC3 I to LC3 II, which is considered as more appropriate than LC3 II alone to reflect the dynamic process of autophagy activity, was also explored. Increased autophagosome formation may result from either increased autophagosome activity or decreased turnover of autophagosome to autolysosome. The autophagy flux, which was performed in our study, could monitor the dynamic process of autophagosome formation and thus accurately reflect the autophagy activity. Furthermore, 2 distinctive types of PRP releasate at 2 different concentrations were also adopted in our study. Even under the circumstances of autophagy increase and autophagy inhibition, the effect of PRP releasate on autophagy activity was further investigated. Therefore, much more solid evidence could be observed in our study.

## 5. Conclusion

Taken together, PRP releasate exerted a beneficial effect in cartilage repair, but autophagy might be independent of the process. These results suggested that PRP was a promising therapy for cartilage repair; however, more studies are warranted to unveil its underlying mechanism.

## Figures and Tables

**Table 1:** Primers for RT-PCR.

Gene name	Forward (5′-3′)	Reverse (5′-3′)
Aggrecan	TCCACATCAGAAGAGCCATAC	AGTCAAGGTCGCCAGAGG
COL2	CTTAGGACAGAGAGAGAAGG	ACTCTGGGTGGCAGAGTTTC
MMP13	AAAGAACATGGTGACTTCTACC	ACTGGATTCCTTGAACGTC
ADAMTS5	TGTGGTGCGCCAAGGCCAAA	CCCTGTGCAGTAGCGGCCAC
GAPDH	CTCAACTACATGGTCTACATGTTCCA	CTTCCCATTCTCAGCCTTGACT

## Data Availability

All data generated or analyzed during this study are included in this article and any additional data are available from the corresponding author upon request.

## List of Abbreviations

PRP: Platelet-rich plasma
OA: Osteoarthritis
IL-1*β*: Interleukin-1 beta
TGF-*β*: Transforming growth factor *β*
PDGF: Platelet-derived growth factor
L-PRP: Leucocyte-rich PRP
P-PRP: Pure PRP
WB: Whole blood
ACD-A: Anticoagulant Citrate Dextrose Solution A
RBCs: Red blood cells
TNF-*α*: Tumor necrosis factor-*α*
ELISA: Enzyme linked immunosorbent assay
DMEM/F-12: Dulbecco's Modified Eagle's Medium/F-12
COL2: Collagen II
MMP: Matrix metalloproteinase
shRNA: Small hairpin RNA
ADAMTS: A disintegrin and metalloproteinase with thrombospondin motifs
TEM: Transmission electron microscopy.
